# Management of Inflammatory Troubles in the Uterine Appendages (Spaying Not Considered)*Read before the Buffalo Obstetrical Society.

**Published:** 1888-12

**Authors:** Marcell Hartwig

**Affiliations:** 38 East Huron Street; Buffalo, N. Y.


### *MANAGEMENT OF INFLAMMATORY TROUBLES IN THE UTERINE APPENDAGES (SPAYING NOT CONSIDERED).**

By MARCELL HARTWIG, M. D., Buffalo, N. Y.

Gentlemen, please consider this paper merely as a medical chat and inducement to exchange views. The invitation to furnish a paper came so late that I had to write without consulting one page of literature.

A highly-interesting subject is this, and the most difficult in gynecology to handle. The palm of success so seldom winks in this field, while, on the other hand, the true physician has all the labor he wishes for. The difficulty we have to contend with is great in the big bulk of cases, requiring lots of ingenuity, patience, and, actually, diplomatic skill. Were it not a fact that the great majority of these inflammatory changes do *not* lead to death, while spaying is much more apt to do so, we would have opportunities to resort to spaying almost every week, with only a moderate amount of gynecological cases in a field of general practice, as is my lot to have to-day. If you take a bird's-eye view of gynecological cases, as they have occurred in your practice during years, and your pathological penchants are similar to

* Read before the Buffalo Obstetrical Society.

mine, you will say, "I am sorry I cannot do spaying with absolute safety, as all other troubles peculiar to women do not amount to a row of pins, the few large tumors excepted."

There was a time in gynecology when Sims's views of mechanical dysmenorrhœa governed the field. Now, as I think, a better understanding has dawned, the sepsis has its full sway; septicemic views (not the view is septic) predominate, I do think, correctly. If you take any intractable case of suffering coming from the female genital organs, sepsis is at the bottom of the case, neurosis comes next, bleeding, new growths, and mechanical difficulties follow in the given order of frequency.

How easy is it to master bleeding; how simple to correct mechanical troubles; how clear, at least, the road to handle new growths; but how beset with uncertainties the septic troubles, the minute they are not entirely confined to outer parts. As soon as the ligaments, or the tubes, or ovaries are involved—and mostly all three, and even their neighborhood, are concerned—the herculean task begins for patient and doctor. The purely neurotic affections are no plaything either, but how scarce. It is often enough that we succeed only because the patient has gone to another doctor.

Here, more than anywhere, the old adage remains true: "An ounce of prevention is worth a pound of cure." Therefore, let us look at the causes of these inflammatory peri-uterine changes.

I have implied already, in my previous statements, that sepsis is their origin, and it is. The two great poisons within this septic field are the gonorrhœic and the putrefacient—how many kinds of bacilli and cocci the latter may contain, not considering. A third poison is the syphilitic; a fourth, the tubercular. The latter two are far less frequently active in the etiology of our cases. Traumatism is accused everywhere of producing troubles in the adnexa, but it is indirectly, by giving septic poisons an avenue. All other poisons of organized nature can produce peri- and para-uterine troubles, perhaps, but I have not seen a single case where I could have accused the poison of small-pox, measles, typhoid, etc., of playing havoc in the adnexa. How easy it would seem now to prevent all tubo-ovarian and ligamental troubles, but what seems easy is, as you all know, well-nigh unsurmountable. You can pull an elephant through the eye of a needle before you can drive the clap out of existence. Syphilis and tuberculosis have the same answer. Putrefaction! Here we have a handle; here we will reap our glory as a profession. Let us enlighten the public, and we will lose practice by preventing disease. Of course, we have to possess the light ourselves. Throw away prejudice before you want to destroy it elsewhere; acquire knowledge before you try to disseminate some. Teach your students to a higher standard. Let us be students ourselves. The traumatism causing adnexal, inflammatory changes is, with few exceptions, the traumatism of timely or untimely birth, and of our operations, big or small ones. Let us have aseptic births and miscarriages.

First, about the latter: there is not a speck of doubt in my mind that the big bulk we meet is purposely induced when syphilis is not the cause. Has experience proven that law and prevailing notions of morality are not sufficient barriers to prevent the daily inducement of miscarriages on the sly, with their ill consequences; then why not drag the object into full light, and thereby prevent lamentable suffering? Were all the miscarriages conducted by regular, well-educated physicians, the amount of parametritic troubles would drop at once to one-half. All that would be necessary would consist in the abolition of laws of mediæval character, which practical viewing shows to be obsolete in the minds of the people. The woman who had an abortion induced on herself, and who considers herself a culprit, I have yet to see. Morality and love of truth are identical. Let us have some truths. For my part, I cannot see a crime in the inducement of abortion the minute the existing law about abortion is abolished; morality laws are not eternal laws. It would be immoral here to induce a woman to self-destruction on account of her husband's death, but it is very moral in India. A duel is immoral here, but not in Germany. What I have said about laws of morality is certainly true about laws in general. No European country has the silly law that attempted suicide is a punishable crime. In Germany, at least, the giving of advice how to prevent conception is not against the criminal law, like here. It is considered a perfectly private affair—why not the inducement of abortion? I think it ought to be considered so. The fetus is accorded rights which I do not see it possess. I cannot but begin to see a right for it after it has severed its connection with the mother. It is a part of her body, and nobody can own anything more than his own body.

And now to the miseries of clandestine abortions. They are expressed simply in one word: septicemia, and death or peri-uterine inflammation as consequences. I do not speak of the misery of clandestine birth for the *child* of avoided abortion, or for the child of a pregnant woman, though I will mention that here in Buffalo, in my estimation, 1,000 babies die every year from diarrhea, because their pregnant mothers have not committed abortion, and their fathers not practiced onanism.

The confinements are the second fruitful source of septicemic ovaritis, salpingitis, para- and perimetritis. Let us have aseptic confinements. How they are possible, you all know, but the public does not, especially the less educated classes. Teach them to avoid midwives. I have, in my recollection, only *one* case of para- and perimetritis in a girl who never had gonorrhea. It came of a fall from a horse. Married, she remained barren. I have seen only one case of syphilitic parametritis, and that case was not, beyond doubt, and never, a tubercular case, though we know that some happen. The gonorrhea I have dismissed somewhat too summarily. Of course, its prevention I cannot discuss, and my expectation I have mentioned, but I have not said that more and more I have been growing to believe with Noeggerath, that it is an incurable disease—that is, sometimes. The discovery of Neisser will settle the question definitely. Until sufficient research has elucidated every point in regard to the longevity of the gonococcus, I shall continue to believe that *some* men who have had the gonorrhea ten years ago can induce it in their wives, and that *some* women who had it ten years ago can develop a gonorrheal parametritis or salpingitis from surviving gonococci, through a favorable occurrence for their transfer from one part of the genital canal to another, say from tube to ovary.

### PATHOLOGY, DIAGNOSIS, PROGNOSIS AND TREATMENT.

The etiology and general knowledge of pathology give us the clue to a diagnosis. Modern tendencies to rely on objective phenomena, and the tendency of the busy practitioner to save time, induce many (the specialists mainly) to proceed too hastily to local examination, as a well-known story illustrates, where the gynecologist seated a girl, ruptured the hymen, and introduced a pessary before the astonished and horrified girl had time to tell that she had a sore toe. The history of a case comes first. The history is frequently sufficient to establish the diagnosis of inflammation in the appendages—I ought to have said mostly. A girl who has every time violently painful menstruation, but who never had a feverish seizure with it, forcing her to bed for a week or more, who never had a violent accident, has no inflammation in the appendages, etc. On the other hand, where a discharge existed in a girl, with, perhaps, painful urination and intensely yellow-greenish spots in the underwear, where afterwards, perhaps, with the next menstruation, which had been painless hitherto, violent colics and high fever or steady pain on the right or left side occurred, which is there most of the time yet, there is a chronic inflammation of the appendage. A woman who had a childbed of eight or ten weeks, and remained barren for the next six years, has probably had inflammation in the adnexa, and so on.

The acute spell of inflammation is somewhat more difficult to recognize by the history alone, if the attack is the first. We receive the impression of peritonitis, which is always there, more or less of it, and we might doubt the origin in the genitalia, and think of perforation of the processus vermiformis if the main intensity of the pain is in the right hypogastrium. The examination does reveal here a good deal less, as in chronic cases, because the great intensity of the pain prevents thorough examination. Only after days the case becomes clear when the pain has lessened; at the start only there, where a distinct cause has been found. I told you in my introduction that I do not intend to write a manual about this disease, or respecting these diseases. I am not reading to medical students, so do not expect me to work the subject systematically. The interesting points—thoughts which crossed my brain concerning these maladies—I intended to bring forth, and so let me jump at once to prognosis and treatment.

The prognosis will fall very much with the form of affection. The acute case requires a prognosis *dubia quond vitam vergens ad bonam* on the first day, or, rather, the minute you can exclude perforation, ileus, and the spread of peritonitis to the upper part of the abdomen. If, after the first morphine injection which you will probably give, the respiration will become abdominal again, especially if the dose did not exceed one-sixth of a grain, the peritoneal symptoms will soon localize near the pelvis, the danger is over—that is, the immediate danger to life—even if a tubo-ovarian pregnancy should have come to bursting. If intense vomiting persists, the probability of the peritonitis generalizing itself is greater, especially, when *facies hippocratica* with cold sweat, a thready and very rapid pulse exists. If the affection is localized from the beginning, the prognosis is more favorable, but easy to make, though a violent chill, where the teeth chatter for half an hour, and, certainly, when such chill repeats itself, ought to make us extremely guarded, even if the pain is localized and peritonitis indistinct. Here we may just meet, and probably do meet, one of the most dangerous forms of general septicemia, or thrombosis in the veins, eventually endocarditis ulcerosa, while the inflammation in the appendages is of secondary consideration. This view is fortified when such chill repeats itself, and no other adequate cause (say an abscess) is found.

There are in the main two affections of the appendages: one leading to chronic thickening of the tissues and adhesions with neighboring organs; another leading to formation of pus. The latter form leaves, after the pus has disappeared one way or another, residues resembling the first. Some may doubt the first form altogether; I do not. It seems to me that the slowness of development of the inflammation produces adhesions before pus could form. Such form seems to occur especially in girls addicted to promiscuous connections. Probably this form is less frequent anyhow. The dissection table would seem to make it the most common, but the histories of those who are dissected are only too often entirely wanting or deficient, while it is perfectly wonderful how much inflammatory deposit can be absorbed in years. I have observed a case where I punctured Douglas' pouch with a trocar, releasing a cup of the stenchiest pus, where, afterwards, a great and bulky amount of thickening remained, where this solid remnant of the exudate reduced itself by half or more in a year, and where, after two years, only a thickening, the size of a walnut, was detectable on one side with careful bimanual palpation. I have observed a uterus of a woman who was barren for years since her first baby, and saw it, I think, after about five years, become movable after it stayed solidly impacted so long.

Our Fellow, Dr. W. W. Potter, is the great champion of a belief which I do not share, that methodical tamponning gives such a result into our hands in a short time. Time is the great element in dispersing those exudates, although tamponning, hot douches and poultices, warm, full salt and mud baths, iodides and general tonics are hastening the process. The treatment of the acute case is that of peritonitis. I have to say one word about it only: opiates are the leaders, ice and antiseptics the principles, generally accepted. The details may vary, but of late a great splurge was made about salty aperients by Tait. His idea is to carry off the septic germs by profuse intestinal discharges. His results, he says, were good. My idea is not in conformity with these views. I do not believe intraperitoneal septic substances can be carried out per anum without perforation of the intestine; certainly not

*every* germ. I do not try to deny Tait's facts, but I doubt the explanation. At all events, this plan may answer after an ovariotomy at the first suspicious symptom. But to give the saline aperient when you are just called to an acute localized peritonitis, I would deem highly imprudent, positively and extremely risking to generalize a local peritonitis. Tait's idea is not even new. Traube has already pleaded for early evacuation to prevent too intimate adhesions and sequential ileus. This I deem reasonable, and I do not wait a week or more before I attempt to move the bowels. Three days are mostly enough. The aperient measures must be mild though—a clysma at first, oil or saline next. Calomel might be retained and cause salivation.

What more do you want about acute cases? That pus must be released as early as positively recognizable, every one knows. Pravaz's needle gives us a chance to proceed much earlier than it was possible years ago, and save the patient much prolongation of her suffering. Do not forget to drain with as *thick* a drain as possible. Follow the needle, and you have a good way of entering the abscess. I do think an instrument would be worth constructing which could be slipped over an aspirator needle and then expanded, or the needle itself could be made to consist of two halves, between which an expanding plug could be inserted to make way finally for the drain. You have not heard me speak about the differential diagnosis as to locality, whether the tube, the broad ligament, the ovary, etc., is inflamed. These distinctions are only possible in chronic cases, and even then only seldom without narcosis. One differential diagnosis is desirable, that is, that from extrauterine pregnancy and its rupture, and from hematocele retro-uterina and peri-uterina. Just here the history is most valuable.

Extrauterine pregnancy will mostly occur where acute and sequential chronic inflammation and a long interval of barrenness has existed. The why lies probably in the fact that previous salpingitis has impaired the regularity of motion of the ciliæ towards the uterus. If the courses have been wanting once or twice, then do not forget of possible extrauterine pregnancy. I am not aware, this minute, that a first pregnancy, without previous pelvic cellulitis, was ever extrauterine. Hematocele is likely to occur just where pelvic cellulitis existed previously by bleeding of ruptured vessels in adhesions, and is almost indistinguishable from ruptured pregnancy if it is intraperitoneal.

Now, about the treatment of chronic pelvic cellulitis. Misery is the patient's and our lot. In regard to treatment, we can distinguish two forms in the main: acute outbursts, and the more or less constant ailment mostly aggravated at the menstrual period. The acute outburst has been spoken of at some length, but, let me say again, requires the treatment of peritonitis. In the beginning, ice on the abdomen and in the vagina when fever exists, and opiates. After a few days, a mild laxative and enema; and when abscess forms, its evacuation as early as possible. Such an evacuation will often enough take place spontaneously before you get a chance, as I have witnessed yesterday. A patient (barren) who is said to have had inflammation of the bowels at the age of eight, whom I have treated a year ago for localized peritonitis, started suddenly and similarly with terrific pain on the nineteenth of September, the region of the left ovary being its seat. Ice and morphine, one-quarter grain of the latter subcutaneously. Three days later oil ricini, an enema, and a warm poultice. Too sensitive for thorough bimanual examination yet. On the twenty-third, spontaneous discharge of very offensive, thin pus, probably per os uteri. Bimanually, without narcosis, nothing elicited. Evidently this was a local peritonitis around the fimbriæ of the Fallopian tube and distension of the latter with pus, which, after the tension had increased sufficiently, emptied itself into and through the uterus. Generally, the gonorrheal cases seem to be tubo-ovarian, the post partum cases intraligamentous; though both may be purely peritoneal, as those from perforations of the bowel mostly are. A perforation of the cecum into the mesocolon can sink its products into the right broad ligament, and break through the vagina, imitating perfectly a parametritis.

The chronic ailments from adhesions and matting together of all the pelvic organs have to be treated symptomatically. Iodine internally, iodol and iodoform per rectal suppository, hot poultices and injections, methodical tampon-pressure, massage by combined manipulation, prolonged warm bath with salt or iron and mud. Drinking lots of mineral and saline waters, and all means of increasing tissue-exchange, are useful. Attention to the digestive functions is valuable, because deposits of fat improve the woman by giving the uterus elastic support; a snugly fitting abdominal belt acts likewise. Any intrauterine manœuvre intended is to be executed with the utmost care and strictest antisepsis, or else a fresh flame might break out among the simmering cinders, as intrauterine overactivity alone has often enough produced pelvic peritonitis as a primary cause. The same reasoning applies to replacing deviations in a patient who has had pelvic peritonitis.

*Anodynes.*—They have to be used most guardedly. Nothing can occur more readily than the creation of morphine habit in those cases. Morphine ought to be abstained from altogether, except in feverish spells of local peritonitis. When there is no fever, morphine should be scrupulously avoided. With chloral the same rule should apply. Here and there I would admit an exception for both in shape of a suppository; at the menstrual period, perhaps, especially on the first day or the day before it, when those sufferers have their worst time. More admissible in suppositories are cocaine, extract of belladonna, hyoscyamus, and cannabis indica. But no anodyne should be used steadily. The rectal suppository is certainly preferable to the vaginal. The latter is almost useless. Intrauterine injections or suppositories would be best if they could be introduced without irritation. The quantity ought not to exceed a grain or two. Cocaine ought to enter them every time, or else you get labor pains without pregnancy. Internally, for exacerbations of pain, Hoffman's drops, chloroform water, tinct. of castoreum, viburnum, and carminatives; hot water even does some good. Very bad cases cannot be cured without spaying, and the worst, a total matting together of all the contents of the pelvis, would die from spaying. Here I show you a sample which I struck accidentally through the kindness of Post-mortem Examiner Dr. Weill. Here a probatory incision would have revealed the hopeless condition, and life might have been spared at least. Modern antisepsis makes the probatory incision well nigh secure. Therefore, we are enabled to give the patient the benefit of the doubt every time.

POST-SCRIPTUM.

Revolution in ethical views is as difficult as any other revolution, but it is bound to come, as sure as the historical revolutions had to crop out. America, the land of the free, seems to me destined to accomplish the task. Practice has actually overtaken already the codified law. Every practitioner knows of dozens of cases of illegally induced abortions. If practitioners of medicine would consider this a first-class crime, would they not bring these crimes to the cognizance of the authorities, or would that not be their highest obligation? But they do not do it, as they would in a case where a husband has poisoned his wife, or in analogous cases.

Why? They have not the moral *conviction* of the righteousness of the law. They merely swim with the prevailing notions in their open utterances, but not in their innermost thoughts.

Who, I ask, is benefited by prosecuting the woman who had committed abortion on herself? Is it our duty to multiply mankind? Have our women to manufacture soldiers? or are the poor girls who made a misstep doomed to carry out, so that wet-nurses shall not be wanting for the rich and lazy?

Is a soul killed? Those who know more—or pretend to know more—about souls than I, say the soul is immortal. Is the law against abortions in existence because the male population wants to have a handle to suppress the female, by endowing her with the care of one more child after she has one-half dozen to take care of?

Frankness compels one to answer: The laws and ethics are relics of barbarian times, where woman was the property of man—merely a convenience, a propagating machine, a vessel, as some old writings called her.

America has elevated the standard of womanhood already above the old country standard in many respects. She will have to do it in this, as it will give her the power to vote. Then, surely, her serfdom will be at an end. If the women had made the laws, abortion would be a word entirely wanting in the criminal code.

Were it not better for the education of the masses, for their well-being in general, if the mothers, especially of the poor, had to take care of a lesser number of children? Who will dare to answer in the negative? Who will dare to deny that the world is better off when a misled (not to speak of a raped) girl has severed her connection with the fetus? That she is better off is not worth while talking, and fetus can be glad certainly. It is very easy to legislate misery into the world, but mighty hard to legislate it out of it. Whoever legislated abortion and prevention of conception to be a crime, has not had to go through labor and nursing—was not the child born out of wedlock.

The physicians are the ones who see mankind's misery closer than anybody else; they will earn the honor of having been the first ones to rise against one of the lies of the century, the laws of abortion, with the war cry: Expunge them!

38 East Huron Street.

